# A Common Variant in the SIAH2 Locus Is Associated with Estrogen Receptor-Positive Breast Cancer in the Chinese Han Population

**DOI:** 10.1371/journal.pone.0079365

**Published:** 2013-11-11

**Authors:** Bo Zhang, Yang Li, Xiaodong Zheng, Xianbo Zuo, Fusheng Zhou, Bo Liang, Jun Zhu, Pan Li, Yantao Ding, Zhonglian Huang, Benzhong Wang, Zhendong Chen

**Affiliations:** 1 Department of Oncology, No. 2 Hospital, Anhui Medical University, Hefei, Anhui, China; 2 School of Life Sciences, Anhui Medical University, Hefei, Anhui, China; 3 Institute of Dermatology and Department of Dermatology, No. 1 Hospital, Anhui Medical University, Hefei, Anhui, China; 4 Department of Dermatology and Venereology, Anhui Medical University, Hefei, Anhui, China; 5 State Key Laboratory Incubation Base of Dermatology, Ministry of National Science and Technology, Hefei, Anhui, China; 6 Department of Galactophore Surgery, No. 1 Hospital, Anhui Medical University, Hefei, Anhui, China; Ohio State University Medical Center, United States of America

## Abstract

**Background:**

Genome-wide association studies (GWAS) have identified many loci associated with breast cancer risk. These studies have primarily been conducted in populations of European descent.

**Objective:**

To determine whether previously reported susceptibility loci in other ethnic groups are also risk factors for breast cancer in a Chinese population.

**Method:**

We genotyped 21 previously reported single nucleotide polymorphisms (SNPs) within a female Chinese cohort of 1203 breast cancer cases and 2525 healthy controls using the Sequenom iPlex platform. Fourteen SNPs passed the quality control test. These SNPs were subjected to statistical analysis for the entire cohort and were further analyzed for estrogen receptor (ER) status. The associations of the SNPs with disease susceptibility were assessed using logistic regression, adjusting for age. The Bonferroni correction was used to conservatively account for multiple testing, and the threshold for statistical significance was P<3.57×10^−3^ (0.05/14).

**Result:**

Although none of the SNPs showed an overall association with breast cancer, an analysis of the ER status of the breast cancer patients revealed that the SIAH2 locus (rs6788895; P = 5.73×10^−4^, odds ratio [OR] = 0.81) is associated with ER-positive breast cancer.

**Conclusion:**

A common variant in the SIAH2 locus is associated with ER-positive breast cancer in the Chinese Han population. The replication of published GWAS results in other ethnic groups provides important information regarding the genetic etiology of breast cancer.

## Introduction

Breast cancer, one of the most common malignancies in women worldwide, is a complex polygenic disorder, and genetic factors play a significant role in its etiology [1]–[3]. To date, genome-wide association studies (GWAS) have reported many susceptibility genes/loci that are associated with breast cancer risk, as reported in the National Human Genome Research Institute catalog (NHGRI GWAS Catalog, available at: www.genome.gov/gwastudies). However, most of these studies enrolled women of European descent, who differ from women of other ethnic groups in certain aspects of their genetic architecture. Additional studies are needed to determine whether the breast cancer susceptibility genes/loci identified in Europeans are also risk factors for breast cancer in the Chinese population.

In 2012, we collaborated with a German research group that had previously validated breast cancer susceptibility genes in German and Chinese cohorts [4]. We evaluated 18 SNPs in 13 susceptibility genes/loci and identified 7 SNPs in 3 loci (ESR1, FGFR2, and TOX3) that were associated with breast cancer in the Chinese population.

In this study, we evaluated susceptibility loci for breast cancer risk that were recently identified in GWAS. In addition, we examined the association of these SNPs with breast cancer estrogen receptor (ER) status.

## Materials and Methods

### Study Population

All 1203 breast cancer cases and 2525 healthy controls (female only) were enrolled based on physician referral through collaborations with multiple hospitals in provinces in central China. All breast cancer patients were diagnosed and categorized according to the tumor-node-metastasis (TNM) breast cancer classification. Clinical information was collected from the affected individuals through a full clinical examination by breast cancer specialists. Additional demographic information was collected from the cases via a structured questionnaire. The ER status of each case was evaluated by examination of breast tissue by biopsy or cytology and immunohistochemical analysis of ER positivity or negativity. These cases consisted of 807 estrogen receptor-positive individuals (67.1%) and 396 estrogen receptor-negative individuals (32.9%). Cases with one or more first-degree relatives with breast or ovarian cancer were considered to have a family history of breast cancer. All Chinese controls were clinically confirmed to be free of breast cancer, other neoplastic diseases, and systemic disorders. Additionally, all controls lacked a family history of neoplastic diseases (including first-, second-, and third-degree relatives). All the cases and controls were female. The characteristics of the study population are presented in Table 1. All participants provided written informed consent. The study was approved by the Ethics Committee of Anhui Medical University and conducted according to the principles of the Declaration of Helsinki.

**Table 1:** Baseline characteristics of breast cancer patients and controls.

Characteristics	Sample
**Cases**	
Sample size	1203
Mean age (years) at onset	48.1±10.4
Mean age (years)	49.7±10.6
No. of ER-positive cases	807
No. of ER-negative cases	396
TNM staging	
Tis	3.60%
T1	21.60%
T2	66.70%
T3	6.60%
T4	1.50%
N0	48.90%
N1	38.10%
N2	11.10%
N3	1.90%
M0	85.90%
M1	14.10%
Familial history of breast cancer (%)	
Familial	7.90%
Sporadic	92.10%
**Controls**	
Sample size	2525
Mean age (years)	50.2±9.6

### SNP selection

Recent GWAS have identified more than 30 different loci associated with breast cancer risk, which can be viewed in the NHGRI GWAS Catalog. Most of these studies enrolled women of European descent. After excluding 18 SNPs in 13 previously reported susceptibility genes/loci [5]–[15] that we evaluated previously [4], 21 new SNPs from recently published GWAS [16]–[26] in European, African and Asian populations were selected for analysis in this study (Table 2). These 21 SNPs represent 20 independent loci present either in genes or intergenic regions.

**Table 2:** The 21 candidate SNPs.

SNP	Chr	Gene	Allele^a^	OR	*P*	Reference	Sample source
rs8170	19p13	*BABAM1*	A/G	1.26	2.3×10^−9^	Nat Genet.2010;42(10):885–892.(16)	European
rs2363956	19p13	*BABAM1*	A/G	0.84	5.5×10^−9^	Nat Genet.2010;42(10):885–892.(16)	European
rs865686	9q31.2	*KLF4*	G/T	0.89	1.8×10^−10^	Natl Cancer Inst.2011;103(5):425–35.(17)	European
rs10411161	19q13.33	*ZNF577*	T/C	1.42	7.1×10^−6^	Hum Genet.2011;130(4):529–37.(18)	European
rs1092913	5p15.2	*ROPNIL*	T/C	1.45	1.9×10^−6^	Hum Genet.2011;130(4):529–37.(18)	European
rs1429142	4q31.23	*EDNRA*	C/T	1.27	3.6×10^−4^	Hum Genet.2011;130(4):529–37.(18)	European
rs1981867	16q23.2	*C16orf61*	A/G	1.22	4.3×10^−4^	Hum Genet.2011;130(4):529–37.(18)	European
rs6723097	Chr2	*CASP8*	A/C	1.16	1.9×10^−4^	Lancet Oncol.2011;12(5):477–488.(19)	European
rs17879961	Chr22	*CHEK2*	C/T	1.52	4.8×10^−8^	Lancet Oncol.2011;12(5):477–488.(19)	European
rs231775	Chr2	*CTLA4*	A/G	1.25	1.6×10^−6^	Lancet Oncol.2011;12(5):477–488.(19)	European
rs17878362	Chr17	*TP53*	16 bp del/insertion	1.15	7.0×10^−3^	Lancet Oncol.2011;12(5):477–488.(19)	European
rs10069690	5p15	*TERT-CLPTMIL*	T/C	1.18	1.0×10^−10^	Nat Genet.2011;43(12):1210–4.(20)	European, African
rs10822013	10q21.2	*ZNF365*	T/C	1.12	5.9×10^−9^	Hum Mol Genet.2011;20(24):4991–9.(21)	Chinese, Japanese
rs2284378	20q11	*RALY*	T/C	1.08	1.3×10^−6^	Hum Mol Genet.2012;21(24):5373–5384.(22)	European
rs17530068	6q14	*RPL17P25-FAM46A*	C/T	1.12	1.1×10^−9^	Hum Mol Genet.2012;21(24):5373–5384.(22)	European
rs9485372	6q25.1	*TAB2*	A/G	0.90	3.8×10^−12^	PLoS Genet.2012;8(2):e1002532.(23)	Chinese, Japanese, Korean
rs7107217	11q24.3	*BARX2*	C/A	1.08	4.6×10^−7^	PLoS Genet.2012;8(2):e1002532.(23)	Chinese, Japanese, Korean
rs13393577	2q34	*ERBB4*	C/T	1.53	8.8×10^−14^	Breast Cancer Res.2012;14(2):R56.(24)	Korean
rs6788895	3q25.1	*SIAH2*	T/G	1.22	9.4×10^−8^	J Hum Genet.2012;57(12):766–71.(25)	Japanese
rs4322600	14q31	*GALC*	A/G	1.18	4.3×10^−6^	Hum Genet.2013;132(1):39–48.(26)	African
rs10510333	3p26	*GRM7*	T/C	1.15	1.5×10^−5^	Hum Genet.2013;132(1):39–48.(2)	African

a Minor allele/major allele.

### Genotyping and quality controls

Genotyping analyses were conducted using the Sequenom MassArray system at the State Key Laboratory Incubation Base of Dermatology, Ministry of National Science and Technology, Hefei, Anhui, China. Genomic DNA was extracted from whole blood or buffy coat samples using FlexiGene® DNA kits (QIAGEN, Hilden, Germany). The DNA quality of all samples was analyzed using a Nanodrop Spectrophotometer ND-2000 (Thermo Scientific, Wilmington, USA), and agarose gel electrophoresis was performed to ensure the genomic integrity of the samples. Approximately 15 ng of genomic DNA was used to genotype each sample. Locus-specific PCR and detection primers were designed using MassARRAY Assay Design 3.0 software (Sequenom, San Diego, USA). Following the manufacturer's instructions, the DNA samples were amplified by multiplex PCR reactions, and the PCR products were subsequently used for locus-specific single-base extension reactions. The resulting products were desalted and transferred to a 384-element SpectroCHIP array. Allele detection was performed using matrix-assisted laser desorption ionization time-of-flight mass spectroscopy (MALDI-TOF MS). The mass spectrograms were analyzed by MassARRAY Typer software (Sequenom). The exclusion criteria for the genotyped SNPs were a call rate of <95%, a minor allele frequency (MAF) of <0.05, and deviation from Hardy-Weinberg equilibrium (HWE, P<0.05) in the controls. Fourteen SNPs passed the quality control test and were subjected to statistical analysis.

### Statistical analysis

The association between the SNPs and disease susceptibility was assessed using logistic regression, adjusting for age. The strength of association was estimated by calculating the odds ratio (OR) with a 95% confidence interval (CI). Hardy-Weinberg equilibrium was assessed using the chi-square test. All statistical analyses were performed with the SPSS 13.0 and Plink 1.07 software packages. In total, 14 SNPs were subjected to statistical analysis. The Bonferroni correction was used to conservatively account for multiple comparisons, and the threshold for statistical significance was P<3.57×10^−3^ (0.05/14).

## Results

To identify genetic variants associated with breast cancer susceptibility in the Chinese population, we selected 21 SNPs from newly published GWAS for a replication study (Table 2). Of the 21 SNPs, 7 were excluded from further analyses because they did not pass the quality control test due to a significant deviation from HWE (rs10411161, rs231775, rs2284378, and rs13393577), a low call rate (rs17879961 and rs17878362), or a low MAF (rs8170). Of the remaining 14 SNPs analyzed in this study, none exceeded the threshold of statistical significance for association (P<3.57×10^−3^) in this cohort (1203 cases, 2525 controls) (Table 3).

**Table 3:** Association between breast cancer susceptibility in the Chinese population and 14 SNPs previously identified by GWAS.

SNP	Chr	Gene	Allele^a^	All women					Estrogen receptor-positive					Estrogen receptor-negative				
				(1203 cases, 2525 controls)					(807 cases, 2525 controls)					(396 cases, 2525 controls)				
				MAF^b^		OR (95% CI)	P	Power	MAF^b^		OR (95% CI)	P	Power	MAF^b^		OR (95% CI)	P	Power
				Cases	Controls				Cases	Controls				Cases	Controls			
rs2363956	19p13	*BABAM1*	A/G	0.3200	0.3303	0.95(0.87∼1.05)	3.12×10^−1^	87	0.3197	0.3303	0.95(0.85∼1.07)	4.27×10^−1^	85	0.3296	0.3303	1.00(0.84∼1.18)	9.68×10^−1^	52
rs865686	9q31.2	*KLF4*	G/T	0.0632	0.0660	0.950.80∼1.14)	5.99×10^−1^	74	0.0657	0.0660	0.99(0.80∼1.24)	9.64×10^−1^	52	0.0587	0.0660	0.88(0.63∼1.23)	4.58×10^−1^	74
rs1092913	5p15.2	*ROPNIL*	T/C	0.3031	0.2958	1.04(0.94∼1.14)	4.63×10^−1^	83	0.3165	0.2958	1.10(0.98∼1.24)	1.11×10^−1^	90	0.2877	0.2958	0.96(0.81∼1.14)	6.58×10^−1^	64
rs1429142	4q31.23	*EDNRA*	C/T	0.3457	0.3335	1.06(0.96∼1.16)	2.37×10^−1^	87	0.3433	0.3335	1.05(0.93∼1.17)	4.63×10^−1^	84	0.3394	0.3335	1.03(0.87∼1.21)	7.56×10^−1^	58
rs1981867	16q23.2	*C16orf61*	A/G	0.3424	0.3407	1.01(0.92∼1.10)	8.68×10^−1^	52	0.3307	0.3407	0.96(0.85∼1.08)	4.56×10^−1^	86	0.3520	0.3407	1.05(0.89∼1.24)	5.53×10^−1^	81
rs6723097	Chr2	*CASP8*	A/C	0.4710	0.4723	0.99(0.91∼1.08)	9.02×10^−1^	58	0.4867	0.4723	1.06(0.95∼1.18)	3.09×10^−1^	87	0.4385	0.4723	0.87(0.75∼1.02)	9.04×10^−2^	93
rs10069690	5p15	*TERT- CLPTMIL*	T/C	0.1802	0.1836	0.98(0.88∼1.09)	6.93×10^−1^	70	0.1769	0.1836	0.96(0.83∼1.10)	5.39×10^−1^	84	0.1927	0.1836	1.06(0.87∼1.30)	5.54×10^−1^	82
rs10822013	10q21.2	*ZNF365*	T/C	0.4788	0.4620	1.07(0.98∼1.17)	1.23×10^−1^	75	0.4778	0.4620	1.07(0.95∼1.19)	2.65×10^−1^	74	0.4749	0.4620	1.05(0.90∼1.23)	5.19×10^−1^	73
rs17530068	6q14	*RPL17P25-FAM46A*	C/T	0.2230	0.2151	1.05(0.94∼1.16)	3.84×10^−1^	85	0.2283	0.2151	1.08(0.95∼1.23)	2.59×10^−1^	87	0.2039	0.2151	0.93(0.77∼1.14)	4.94×10^−1^	84
rs9485372	6q25.1	*TAB2*	A/G	0.4127	0.4340	0.92(0.84∼1.00)	4.87×10^−2^	78	0.4083	0.4340	0.90(0.80∼1.01)	6.74×10^−2^	78	0.4190	0.4340	0.94(0.80∼1.10)	4.50×10^−1^	74
rs7107217	11q24.3	*BARX2*	C/A	0.3562	0.3374	1.09(0.99∼1.19)	7.06×10^−2^	77	0.3565	0.3374	1.09(0.97∼1.22)	1.56×10^−1^	75	0.3226	0.3374	0.94(0.79∼1.11)	4.34×10^−1^	74
rs6788895	3q25.1	*SIAH2*	T/G	0.3489	0.3759	0.89(0.81∼0.97)	9.96×10^−3^	97	0.3289	0.3759	0.81(0.72∼0.92)	5.73×10^−4^	99	0.3725	0.3759	0.99(0.84∼1.16)	8.61×10^−1^	58
rs4322600	14q31	*GALC*	A/G	0.0586	0.0625	0.93(0.78∼1.12)	4.51×10^−1^	84	0.0580	0.0625	0.92(0.73∼1.17)	5.03×10^−1^	84	0.0629	0.0625	1.01(0.73∼1.39)	9.73×10^−1^	52
rs10510333	3p26	*GRM7*	T/C	0.1767	0.1854	0.94(0.84∼1.05)	3.02×10^−1^	85	0.1725	0.1854	0.92(0.79∼1.06)	2.39×10^−1^	88	0.2039	0.1854	1.13(0.93∼1.37)	2.35×10^−1^	87

a Minor allele/major allele.

b Minor allele frequency.

In addition, the associations of these 14 SNPs with ER status were evaluated (Table 3). The association of rs6788895 with ER-positive breast cancer was statistically significant (P = 5.73×10^−4^, OR = 0.81, 95% CI = 0.72–0.92). No other SNPs were significantly associated with ER-positive or ER-negative breast cancer in this Chinese population.

## Discussion

In this case-control study of women of Chinese descent, 21 SNPs from newly published GWAS of breast cancer were evaluated. Among these SNPs, 14 passed the quality control test and were subjected to further analysis. The statistical power calculations showed that 7 SNPs (rs2363956, rs1092913, rs1429142, rs17530068, rs6788895, rs4322600 and rs10510333) could be detected with ≥80% power, whereas 7 other SNPs (rs865686, rs1981867, rs10822013, rs9485372, rs7107217, rs6723097 and rs10069690) were detected with low power (<80%). We confirmed that the 7 SNPs that had ≥80% power to detect an association were not associated with breast cancer. However, these SNP loci cannot be ruled out as risk factors for breast cancer due to the potential for different functional variants in Chinese and other ethnic populations and/or different linkage disequilibrium (LD) patterns between the markers and hidden functional variants. Genotyping other known SNPs in these regions using HapMap data will facilitate the elucidation of potential ethnicity-related disparities in the association of these previously reported loci with breast cancer in different populations and provide a better understanding of the genetic basis of breast cancer. Among the SNPs detected with low power, 4 were previously reported as being associated with breast cancer in a European population (rs865686, rs1981867, rs6723097 and rs10069690), whereas the other 3 (rs10822013, rs9485372 and rs7107217) were previously reported as being associated with breast cancer in an Asian population. We could not confirm that the low power SNPs lacked an association with breast cancer because we might lack the power to detect a true association. Larger sample sizes will help increase the power and confirm whether these SNPs are associated with breast cancer in the Chinese Han population.

Although all SNPs lacked an overall association with breast cancer, rs6788895 was found to be associated with ER-positive breast cancer and was previously reported to be associated with hormonal receptor-positive breast cancer risk in a Japanese population. The current study is the first to show that rs6788895 is also correlated with ER-positive breast cancer in a Chinese population. Overall, the results of this study demonstrate the association of GWAS-identified SNPs with breast cancer risk in a Chinese population.

The rs6788895 SNP is located in the intronic region of the Siah E3 ubiquitin protein ligase 2 (SIAH2) gene on chromosome 3q25.1. This gene encodes a protein that is a member of the seven in absentia homolog family and is involved in the ubiquitination and proteasome-mediated degradation of specific proteins. Many studies have shown that SIAH2 is closely related to breast cancer. Elgazzar et al. reported that SIAH2 induces the degradation of many proteins, including POU2AF1, PML, NCOR1, DCC, and BAG1. These proteins were reported to have a relationship with breast cancer [25]. In addition, growing evidence indicates that SIAH2 plays a critical role in the hypoxia response [27]. Hypoxia is a pivotal driver of breast tumor progression that leads to the transcription of several suites of genes involved in angiogenesis, cell survival, cell proliferation, and an enhanced metastatic phenotype, all of which are advantageous for neoplastic cells [28]. Growing evidence also indicates an important function for SIAH2 in tumor development and progression based on a study of mammary tumors [27]. Moreover, SIAH2 is closely related with the ER status of breast cancer. SIAH2 sequence analysis revealed an estrogen response element (CAGGTCANNNTGACCTG) [29] in the intron between exons 1 and 2. Estrogen has been reported to induce the expression of SIAH2 in ER-positive breast cancer cell lines [30], [31]. SIAH2 is an ER-positive epithelial breast cancer subtype gene and is strongly associated with ER levels [32], [33]. In addition, Sarkar et al. identified a Src tyrosine kinase/SIAH2 E3 ubiquitin ligase pathway that regulates the expression of the CEBPD tumor suppressor and contributes to the transformation of breast tumor cells [34].

In conclusion, in this Chinese population-based case-control study, a common variant in the SIAH2 locus associated with ER-positive breast cancer was identified. This result demonstrates for the first time that the association between the SIAH2 locus and breast cancer detected previously in the Japanese population is also present in the Chinese population. Future fine mapping and functional analysis studies are warranted to investigate the precise role of SIAH2 in the pathogenesis of breast cancer.
